# The Effect of Aluminum Exposure on Maternal Health and Fetal Growth in Rats

**DOI:** 10.7759/cureus.31775

**Published:** 2022-11-22

**Authors:** Mohammed H Badawoud, Gamal Abdel-aziz, Magdy M El-Fark, Hassan M Badawoud

**Affiliations:** 1 Department of Anatomy, Faculty of Medicine, King Abdulaziz University, Jeddah, SAU; 2 Department of Anatomy, Faculty of Medicine, Suez Canal University, Suez, EGY

**Keywords:** dose, toxicity, fetuses, pregnancy, aluminum

## Abstract

Background

This study was performed on female rats to study the effect of oral administration of low dose versus high dose of aluminum chloride (AlCl3) during the period of organogenesis on the maternal and fetal growth parameters.

Methods

In this study, female mature nulliparous Sprague-Dawley albino rats were used. After mating and confirmation of pregnancy, successfully mated females were divided into three groups (six rats each): control group, low-dose (LD) AlCl3 group, and high-dose (HD) AlCl3 group. The rats were sacrificed at gestational day 20 (GD20) when the liver and kidneys were excised and weighed. Also, the gravid uterine horns were excised and weighed, the placentae and fetuses were extracted and weighed, and fetal growth parameters were assessed.

Results

Maternal AlCl3 exposure produced an increase in preimplantation losses and resorptions in LD and HD AlCl3 groups. Consequently, there was a decrease in the number of corpora lutea, total implantations, live fetuses, and litter size. Also, the body weight gain, gravid uterine, placental and maternal liver, and maternal kidney weights of both AlCl3-treated groups were significantly reduced in comparison with the control group. There was a statistically significant reduction in fetal biparietal diameter (BPD), head length (HL), crown-rump length (CRL), and fetal body weight. All the above changes were dose-dependent, being more evident with the high dose of AlCl3.

Conclusion

AlCl3 exposure during pregnancy results in different degrees of adverse effects on maternal weight gain and fetal growth and organ parameters, which followed a dose-dependent manner.

## Introduction

Aluminum (Alu) is considered as one of the most widely used metals in the environment. It is used in daily life in a manner that leads to easy exposure for human beings [1].

Exposure to Alu has been implicated in a number of human pathologies and intoxication of health, which have been increasingly upsetting in recent years. Alu is known to have many hazardous organ effects such as renal failure, dementia, parkinsonism, Alzheimer’s disease, and amyotrophic lateral sclerosis [2-4].

Alu is being used extensively in our contemporary life, where it is widely used in the treatment of drinking water, in addition to the manufacture of Alu-contained compounds, drugs (e.g., phosphate binders, antacids, vaccines, buffered aspirins, parenteral fluids, and allergen injection), and cosmetics [1,2,5]. Alu food contamination may increase due to the constant use of aluminum cans, foil, and vessels for preparing and storing food where this metal leaches from these containers depending on the type of food being packed or stored [6].

Hence, Alu exposure is mainly due to food intake and water drinking in addition to the use of personal care products and cookware. Drugs, cosmetics, vaccines, inhaled fumes, and particles from occupational exposures can also increase the chance of Alu toxicity. Furthermore, a higher level of exposure to Alu leads to its poor excretion, causing its accumulation in body tissues [1,2,5].

Previous studies showed that Alu had a toxic effect on the development and growth of fetuses and offspring in animals and humans. Alu exposure during pregnancy was found to result in growth retardation, deaths, resorptions, soft tissue abnormalities, and developmental toxicity syndrome in rats and mice [2,7]. Alu was reported to induce neurotoxic effects, hydronephrosis, endocrine disruption, and reproductive toxicity [1,2]. However, controversy and conflicting observations on the toxic effects of Alu showed the need for further studies as some investigations did not report embryotoxic effects for Alu [8].

During the early period of pregnancy, women may use large amounts of Alu-containing antacids as they may have gastric symptoms that may result in increased Alu intake, which in turn considerably increases Alu plasma levels in pregnant females [9]. It was reported that Alu can pass through the placenta to fetuses [10].

Pregnant women may be exposed to untoward levels of Alu compounds; therefore, it is important to investigate the toxic effects of Alu on fetuses.

Our study was conducted on female rats to study the effect of oral administration of low and high doses of Alu during the period of organogenesis on maternal, reproductive, and fetal parameters.

## Materials and methods

This project was approved by the ethics committee of the Faculty of Medicine of King Abdulaziz University (approval number: D/34/1364).

Reagents

The aluminum chloride (AlCl3) powder, distilled water, and Bouin’s solution used in this study were obtained from Sigma Chemicals Co. (St. Louis, MO, USA).

Animals

Virgin female mature Sprague-Dawley albino rats (8-10 weeks old) were used in this investigation. The animals were obtained from King Fahd Medical Research Center. A microscopic examination of the vaginal smear was performed to confirm pregnancy after matting; when it was positive, it was considered gestational day 0 (GD0).

Eighteen pregnant female rats were divided into three groups (six rats each) as follows: control group, which received 2 mL distilled water using an intragastric tube from GD6 to GD15; low-dose (LD) AlCl3 group, which received 173 mg [11] (1/10 of LD50) 22 of AlCl3/kg body weight/day, one dose daily at 9 am from GD6 to GD15; and high-dose (HD) AlCl3 group, which received 345 mg [11] (1/20 of LD50) 22 of AlCl3/kg body weight/day, one dose daily at 9 am from GD6 to GD15.

AlCl3 (345 mg) was dissolved in 10 mL of distilled water, and treatment was given using an intragastric tube to all study groups.

Examination of pregnant female rats

Pregnant rats were anesthetized on GD20 when the gravid uterine horns were obtained and weighed. The ovaries and uteruses were inspected to estimate the number of implantation sites, corpora lutea, and resorptions (embryonic death). All placentae and fetuses were obtained from the pregnant rats and weighed.

Also, the position and number of viable fetuses were detected and counted. The preimplantation loss was estimated as follows: (number of corpora lutea - number of implantations) × 100/number of corpora lutea.

Evaluation of fetuses

After exposing the fetuses and placentas, each fetus and its placenta were removed and weighed. Digital Vernier caliper was used to estimate the head length (HL), biparietal diameter (BPD), and fetal crown-rump length (CRL). Additionally, the absolute weight, actual organ weight, and relative weight, the organ weight/total weight × 100, were recorded for fetal kidneys and livers.

Statistical analysis

The Statistical Package for the Social Sciences (SPSS) (IBM SPSS Statistics, Armonk, NY, USA) for Windows was used to analyze the results. The results were presented as mean±standard deviation (SD). One-way analysis of variance (ANOVA) and the Bonferroni test were used to compare the different groups. The difference was considered significant if P-values were <0.05.

## Results

Effect of AlCl3 on reproductive measures

**Figure 1:**
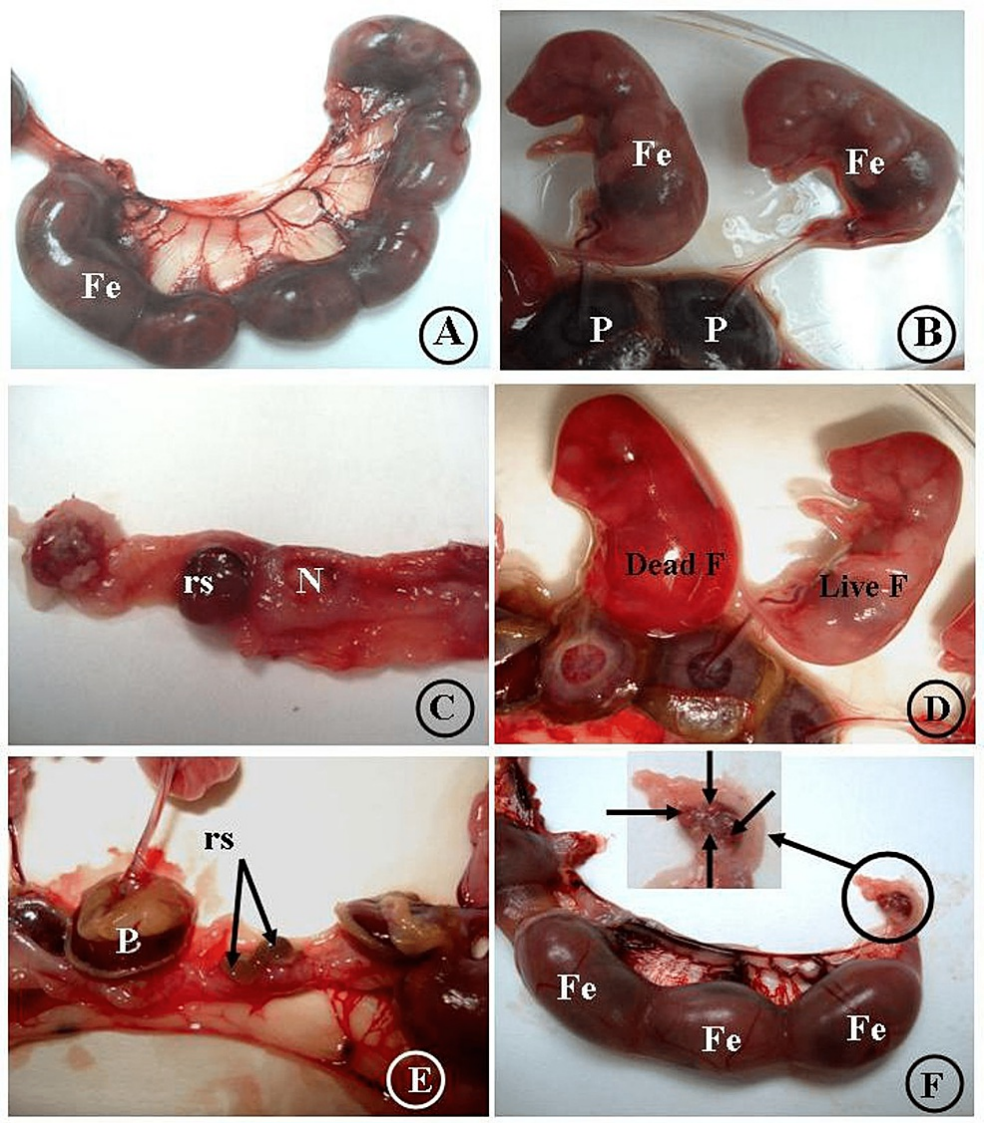
Uterine horn in the control group (A and B) with fetuses (Fe) and their placentas (P). Both LD AlCl3 (C) and HD AlCl3 (D, E, and F) groups show normal sites of implantation (N), normal placenta (P), and sites of resorption (rs). Notice the dead fetus in (D). There were four corpora lutea with only three fetuses (one preimplantation loss) (F). Fe: fetus, P: placenta, N: normal site of implantation, rs: site of resorption

It was noticed that the number of preimplantation losses in both doses of AlCl3 was significantly increased when compared to the control group, while the number of total implantations, corpora lutea, and litter size were decreased when compared to the control group (Table 1 and Figure 1).

**Table 1:** Effect of AlCl3 on reproductive parameters. Results are shown as mean±standard deviation (n=6). A difference of P<0.05 was considered significant. ^a^P<0.05 versus control group ^b^P<0.01 versus control group ^c^P<0.01 versus LD AlCl3 group The Bonferroni test was used to compare the different groups. *Significant difference between groups (P<0.05) **Significant difference between groups (P<0.01) AlCl3: aluminum chloride, LD AlCl3: low-dose AlCl3, HD AlCl3: high-dose AlCl3

Groups	Corpora lutea	Total implantation*	Preimplantation loss	Litter size**
Control	9.5±1.23	9.33±1.03	0.17±0.04	9.17±0.75
LD AlCl3	8.67±0.82^a^	8.17±0.41^a^	0.50±0.08^b^	7.83±0.75^b^
HD AlCl3	8.83±0.98^a^	8.00±0.89^a^	0.83±0.07^b,c^	7.17±1.17^b^

Effect of AlCl3 on pregnant rat and placental weight

It was noticed that in the LD AlCl3 group, there was a significant reduction in all measured parameters, including body weight gain, gravid uterine weight, placental weight, absolute right (Rt) kidney weight, absolute left (Lt) kidney weight, and relative Lt kidney weight, when compared to the control group. In the HD AlCl3 group, there was a dramatic reduction of all measured parameters, including body weight gain, final body weight, gravid uterine weight, placental weight, absolute liver weight, relative liver weight, absolute Rt kidney weight, relative Rt kidney weight, absolute Lt kidney weight, and relative Lt kidney weight; this reduction was statistically significant when compared to the control group.

It was found that there was a statistically significant reduction in some parameters measured in the HD AlCl3 group, including final body weight, body weight gain, gravid uterine weight, and placental weight, when compared with the LD AlCl3 group.

Also, there was a significant difference between the studied groups for final body weight, body weight gain, gravid uterine weight, placental weight, absolute liver weight, relative liver weight, absolute Rt kidney weight, relative Rt kidney weight, absolute Lt kidney weight, and relative Lt kidney weight (Table 2 and Table 3).

**Table 2:** Effect of AlCl3 on pregnant rat weight parameters and placental weight. Results are shown as mean±standard deviation (n=6). A difference of P<0.05 was considered significant. ^a^P<0.01 versus control group ^b^P<0.001 versus control group ^c^P<0.01 versus LD AlCl3 group ^d^P<0.001 versus LD AlCl3 group The Bonferroni test was used to compare the different groups. *Significant difference between and within groups (P<0.001). AlCl3: aluminum chloride, LD AlCl3: low-dose AlCl3, HD AlCl3: high-dose AlCl3

Groups	Body weight gain (gm)* (n=6)	Gravid uterine weight (gm)* (n=6)	Placental weight (gm)*
Control	108.67±5.61	58.36±3.45	0.63±0.05 (n=55)
LD AlCl3	91.34±7.45^a^	49.30±4.09^a^	0.52±0.11^b^ (n=47)
HD AlCl3	65.48±5.68^b,d^	35.48±3.04^b,d^	0.42±0.07^b,d^ (n=43)

**Table 3:** Effect of AlCl3 on pregnant rat liver and kidney weights. Results are shown as mean±standard deviation (n=6). A difference of P<0.05 was considered significant. ^a^P<0.05 versus control group ^b^P<0.01 versus control group ^c^P<0.001 versus control group The Bonferroni test was used to compare the different groups. *Significant difference between groups (P<0.05) **Significant difference between groups (P<0.01) ***Significant difference between groups (P<0.001) AlCl3: aluminum chloride, Rt: right, Lt: left, LD AlCl3: low-dose AlCl3, HD AlCl3: high-dose AlCl3

Groups	Absolute liver weight (gm)**	Relative liver weight (gm)*	Absolute Rt kidney weight (gm)**	Relative Rt kidney weight (gm)*	Absolute Lt kidney weight (gm)***	Relative Lt kidney weight (gm)*
Control	10.90±0.53	4.85±0.20	0.98±0.06	0.44±0.04	0.92±0.05	0.41±0.03
LD AlCl3	9.92±0.52	4.67±0.20	0.80±0.15^a^	0.38±0.06	0.73±0.11^b^	0.34±0.05^a^
HD AlCl3	8.87±0.95^c^	4.42±0.33^a^	0.71±0.11^b^	0.36±0.05^a^	0.67±0.08^c^	0.33±0.04^a^

Effect of AlCl3 on fetal growth parameters

**Figure 2:**
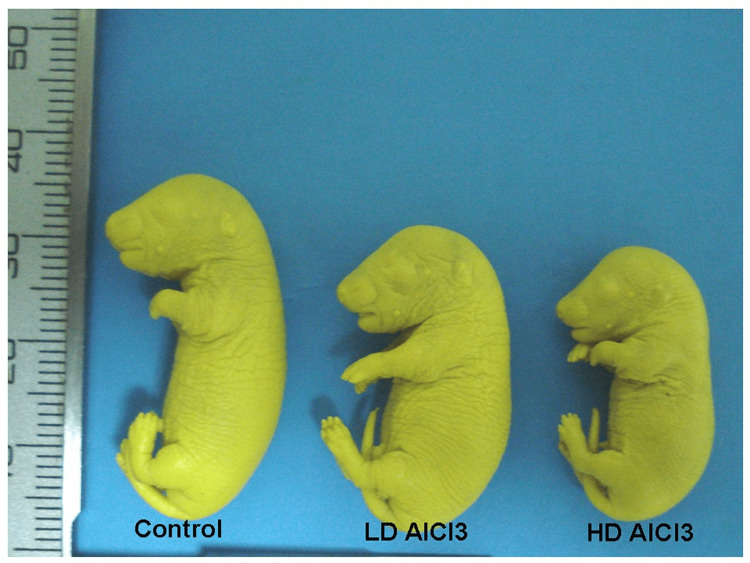
Comparison of the crown-rump length between fetuses representing control, LD AlCl3, and HD AlCl3 groups (fixed in Bouin’s solution). AlCl3: aluminum chloride, LD AlCl3: low-dose AlCl3, HD AlCl3: high-dose AlCl3

It was noticed that in the LD AlCl3 group, all fetal growth parameters, including fetal body weight, crown-rump length, head length, and biparietal diameter, were significantly lower than in the control group. In the HD AlCl3 group, there was a dramatic reduction of all measured fetal growth parameters, including fetal body weight, crown-rump length, head length, and biparietal diameter; this reduction was statistically significant when compared to the control group. Also, these parameters were significantly reduced when compared with the LD AlCl3 group. It was also found that there was a significant difference between and within groups (control, LD AlCl3, and HD AlCl3 groups) for fetal body weight, crown-rump length, head length, and biparietal diameter (Table 4 and Figure 2).

**Table 4:** Effect of AlCl3 on fetal growth parameters. Results are shown as mean±standard deviation (n=6). A difference of P<0.05 was considered significant. ^a^P<0.05 versus control group ^b^P<0.001 versus control group ^c^P<0.0001 versus control group ^d^P<0.001 versus LD AlCl3 group The Bonferroni test was used to compare the different groups. *Significant difference between groups (P<0.001) AlCl3: aluminum chloride, LD AlCl3: low-dose AlCl3, HD AlCl3: high-dose AlCl3

Groups	Fetal body weight (gm)*	Crown-rump length (cm)*	Head length (cm)*	Biparietal diameter (cm)*
Control (55)	2.85±0.24	3.12±0.14	1.30±0.06	0.71±0.05
LD AlCl3 (47)	2.48±0.58^b^	2.89±0.27^b^	1.22±0.07^b^	0.68±0.05^a^
HD AlCl3 (43)	1.80±0.44^c,d^	2.62±0.33^b,d^	1.16±0.09^b,d^	0.63±0.06^b,d^

## Discussion

Because Alu is present in a large amount in man-made products and nature, the human cumulative daily intake of Alu varies broadly in a way that it is difficult to be estimated [12]. Pregnant women may ingest large amounts of Alu through consuming Alu-containing antacids or exposure to high environmental levels of Alu, which might increase their plasma Alu level. This high level of Alu may pass through the placenta or milk to fetuses or offspring [13].

Previous studies suggested that the exposure of pregnant females to Alu imposes a high risk to their health as well as to the development and health of their fetuses and children. These studies have demonstrated that during pregnancy, the intake of high doses of antacids increased considerably its Alu plasma concentration in mothers. They also reported that Alu can be transferred to fetuses through the placenta [14].

In this study, two doses of Alu were used to investigate the toxic effect of Alu intake during pregnancy on maternal and fetal parameters. The results of our study showed that Alu administration to pregnant rats gave rise to adverse effects in mothers and their fetuses, which were dose-dependent. Regarding the mother rats, this was evident from the decrease in body weight gain and decreased absolute and relative liver and kidney weights and gravid uterine and placental weights when compared to the control group. A previous study reported similar results regarding maternal weight gain in female rats who received Alu [2]. Accordingly, it has been stated that orally administered Alu is absorbed systemically, leading to a more accumulation of Alu in target tissues such as the kidney and liver, which accumulate Alu over a wide range of exposure levels and so are probably the most susceptible organs to its effects [15].

Also, the findings of the present study showed that Alu has both embryotoxic and fetotoxic effects as it increased the number of resorptions, preimplantation losses, and dead fetuses with a decrease in the total numbers of implantation sites and corpora lutea, litter sizes, and live fetuses in Alu-treated groups. Also, high-dose Alu-treated groups were significantly different from low-dose Alu-treated groups in most of the above parameters. Accordingly, Lin et al. [16] observed a dose-dependent relationship between Alu intake and intrauterine growth retardation in mice and suggested that excessive Alu ingestion during pregnancy may be one of the risk factors contributing to congenital neural tube defects and perinatal deaths. Also, in agreement with our findings, many previous studies that used different doses of Alu have shown that Alu was both embryotoxic and fetotoxic, resulting in growth retardation, increased mortality, increased resorptions, multiple congenital malformations, a variety of adverse reproductive outcomes, and alteration in neuromotor maturation in exposed animals [1,2,10,17].

However, in disagreement with our results, some earlier studies have reported discrepant information. It has been reported by Gomez et al. [18] that when Alu (Al OH3) is given by gavage at different doses to pregnant rats from GD6 to GD15, there was non-observed significant maternal or developmental toxicity at any Alu dose level. This discrepancy may be due to the usage of low Alu doses.

The present investigation showed that the Alu intake of pregnant rats resulted in a decrease in the number of fetuses and decreased mean values of fetal growth parameters (crown-rump length, body weight, biparietal diameter, and head length). These effects were more significant with a higher dose of Alu when compared with a lower dose of Alu and control values. These toxic effects may be caused by the increased concentration of Alu in the placenta and fetuses [19]. Accordingly, previous studies on the effects of Alu intake during pregnancy on embryos found that there were decreased fetal numbers and increased fetal death. Also, these studies showed that neonates, even if they were born healthy and without any obvious problems, were likely to have growth retardation [2,20,21]. Moreover, data from the study of Kamalov et al. [22] showed that rats exposed to Alu have dose- and time-dependent damage to their lymphocyte and thymocyte plasma membranes.

The human reproductive system may be affected by Alu exposure. Alu was reported to reduce sperm count and motility and increase abnormal spermatozoa [2]. In female mice, Alu accumulates in the ovary, which could damage the ovarian structure [11].

Alu causes a transient disturbance to estrous cycle regularity in female rats but does not develop into reproductive toxicity [11]. It was reported that female employees at an Alu smelter had more congenital malformations than they did during their earlier working periods when they did not have access to Alu and reported that Alu exposure has toxic effects on the reproductive system [23]. It was reported that the Alu level was elevated in the ovarian and uterine tubes of adult female rats after oral administration of Al sulfate, and the tissue levels were correlated to Alu dosages [11]. Wang et al. [24] suggested that Alu exposure affected the secretory activity of the ovary and decreased follicle-stimulating hormone (FSH) and luteinizing hormone (LH) levels in the rat serum. These evidence demonstrate that Alu is a potential risk for female infertility.

The fund obtained for this study did not allow us to do a further investigation such as histopathological evaluation of the fetal brain and organs. However, we hope to obtain another fund soon to perform these studies.

## Conclusions

This study demonstrates that Alu exposure during pregnancy has different degrees of adverse effects on the growth parameters of both mothers and fetuses and their body organs, which follows a dose-dependent manner.

## References

[1] Tietz T, Lenzner A, Kolbaum AE (2019). Aggregated aluminium exposure: risk assessment for the general population. Arch Toxicol.

[2] Igbokwe IO, Igwenagu E, Igbokwe NA (2019). Aluminium toxicosis: a review of toxic actions and effects. Interdiscip Toxicol.

[3] Rondeau V (2002). A review of epidemiologic studies on aluminum and silica in relation to Alzheimer's disease and associated disorders. Rev Environ Health.

[4] Kumar V, Gill KD (2009). Aluminium neurotoxicity: neurobehavioural and oxidative aspects. Arch Toxicol.

[5] Krupińska I (2020). Aluminium drinking water treatment residuals and their toxic impact on human health. Molecules.

[6] Ertl K, Goessler W (2018). Aluminium in foodstuff and the influence of aluminium foil used for food preparation or short time storage. Food Addit Contam Part B Surveill.

[7] Colomina MT, Esparza JL, Corbella J, Domingo JL (1998). The effect of maternal restraint on developmental toxicity of aluminum in mice. Neurotoxicol Teratol.

[8] Agrawal SK, Ayyash L, Gourley CS, Levy J, Faber K, Hughes CL Jr (1996). Evaluation of the developmental neuroendocrine and reproductive toxicology of aluminium. Food Chem Toxicol.

[9] Skarica B (2018). Effectiveness of manual treatment on pregnancy symptoms: usefulness of manual treatment in treating pregnancy symptoms. Med Arch.

[10] Yokel RA (2020). Aluminum reproductive toxicity: a summary and interpretation of scientific reports. Crit Rev Toxicol.

[11] Fu Y, Jia FB, Wang J (2014). Effects of sub-chronic aluminum chloride exposure on rat ovaries. Life Sci.

[12] Klotz K, Weistenhöfer W, Neff F, Hartwig A, van Thriel C, Drexler H (2017). The health effects of aluminum exposure. Dtsch Arztebl Int.

[13] Willhite CC, Karyakina NA, Yokel RA (2014). Systematic review of potential health risks posed by pharmaceutical, occupational and consumer exposures to metallic and nanoscale aluminum, aluminum oxides, aluminum hydroxide and its soluble salts. Crit Rev Toxicol.

[14] Sharma P, Mishra KP (2006). Aluminum-induced maternal and developmental toxicity and oxidative stress in rat brain: response to combined administration of Tiron and glutathione. Reprod Toxicol.

[15] (2008). Agency for Toxic Substances and Disease Registry: Toxicological profiles. http://www.atsdr.cdc.gov/toxprofiles/tp.asp?id=191&tid=34‎.

[16] Lin B, Zhang R, Zhu S (1997). [Studies on teratogenic effects of aluminum on intra uterine fetal development in mice]. Zhonghua Yu Fang Yi Xue Za Zhi.

[17] Caito S, Aschner M (2015). Neurotoxicity of metals. Handb Clin Neurol.

[18] Gomez M, Bosque MA, Domingo JL, Llobet JM, Corbella J (1990). Evaluation of the maternal and developmental toxicity of aluminum from high doses of aluminum hydroxide in rats. Vet Hum Toxicol.

[19] Sharma P, Shah A, Shukla S (2002). Protective effect of Tiron (4,5-dihydroxybenzene-1,3-disulfonic acid disodium salt) against beryllium-induced maternal and fetal toxicity in rats. Arch Toxicol.

[20] Ochmański W, Barabasz W (2000). [Aluminum--occurrence and toxicity for organisms]. Przegl Lek.

[21] Nehru B, Bhalla P (2006). Reversal of an aluminium induced alteration in redox status in different regions of rat brain by administration of centrophenoxine. Mol Cell Biochem.

[22] Kamalov J, Carpenter DO, Birman I (2011). Cytotoxicity of environmentally relevant concentrations of aluminum in murine thymocytes and lymphocytes. J Toxicol.

[23] Sakr CJ, Taiwo OA, Galusha DH (2010). Reproductive outcomes among male and female workers at an aluminum smelter. J Occup Environ Med.

[24] Wang N, She Y, Zhu Y (2012). Effects of subchronic aluminum exposure on the reproductive function in female rats. Biol Trace Elem Res.

